# Micrornas in prostate cancer: an overview

**DOI:** 10.18632/oncotarget.16933

**Published:** 2017-04-07

**Authors:** Daniela Vanacore, Mariarosaria Boccellino, Sabrina Rossetti, Carla Cavaliere, Carmine D'Aniello, Rossella Di Franco, Francesco Jacopo Romano, Micaela Montanari, Elvira La Mantia, Raffaele Piscitelli, Flavia Nocerino, Francesca Cappuccio, Giovanni Grimaldi, Alessandro Izzo, Luigi Castaldo, Maria Filomena Pepe, Maria Gabriella Malzone, Gelsomina Iovane, Gianluca Ametrano, Paola Stiuso, Lucio Quagliuolo, Daniela Barberio, Sisto Perdonà, Paolo Muto, Maurizio Montella, Piera Maiolino, Bianca Maria Veneziani, Gerardo Botti, Michele Caraglia, Gaetano Facchini

**Affiliations:** ^1^ Progetto ONCONET2.0, Linea progettuale 14 per l'implementazione della Prevenzione e Diagnosi Precoce del Tumore alla Prostata e Testicolo, Regione Campania, Italy; ^2^ Department of Biochemistry, Biophysics and General Pathology, University of Campania “L. Vanvitelli” Naples, Naples, Italy; ^3^ Division of Medical Oncology, Department of Uro-Gynaecological Oncology, Istituto Nazionale Tumori ‘Fondazione G. Pascale’, IRCCS, Naples, Italy; ^4^ Department of Onco-Ematology Medical Oncology, S.G. Moscati Hospital of Taranto, Taranto, Italy; ^5^ Division of Medical Oncology, A.O.R.N. dei COLLI “Ospedali Monaldi-Cotugno-CTO”, Napoli, Italy; ^6^ Radiation Oncology, Istituto Nazionale per lo Studio e la Cura dei Tumori ‘Fondazione Giovanni Pascale’, IRCCS, Napoli, Italy; ^7^ Department of Molecular Medicine and Medical Biotechnologies, University of Naples “Federico II”, Naples, Italy; ^8^ Pathology Unit, Istituto Nazionale Tumori “Fondazione G. Pascale”, IRCCS, Naples, Italy; ^9^ Pharmacy Unit, Istituto Nazionale Tumori, Istituto Nazionale Tumori-Fondazione G. Pascale, Naples, Italy; ^10^ Epidemiology Unit, Istituto Nazionale per lo Studio e la Cura dei Tumori ‘Fondazione Giovanni Pascale’, IRCCS, Napoli, Italy; ^11^ Psicology Unit, Istituto Nazionale per lo Studio e la Cura dei Tumori ‘Fondazione Giovanni Pascale’, IRCCS, Napoli, Italy; ^12^ Division of Urology, Department of Uro-Gynaecological Oncology, Istituto Nazionale Tumori ‘Fondazione G. Pascale’, IRCCS, Naples, Italy; ^13^ Scientific Directorate, Istituto Nazionale Tumori ‘Fondazione G. Pascale’, IRCCS, Naples, Italy

**Keywords:** microRNAs, prostate cancer, biomarkers, oncogenic miRNAs, tumor suppressor miRNAs

## Abstract

Prostate cancer is the second highest cause of cancer mortality after lung tumours. In USA it affects about 2.8 million men and the incidence increases with age in many countries. Therefore, early diagnosis is a very important step for patient clinical evaluation and for a selective and efficient therapy. The study of miRNAs' functions and molecular mechanisms has brought new knowledge in biological processes of cancer. In prostate cancer there is a deregulation of several miRNAs that may function as tumour suppressors or oncogenes. The aim of this review is to analyze the progress made to our understanding of the role of miRNA dysregulation in prostate cancer tumourigenesis.

## INTRODUCTION

The most widely type of solid tumor in men is prostate cancer (PCa). Prostate cancer is the secondary highest cause of malignancy mortality, behind lung neoplasms, in developed occidental countries. It is assessed that prostate cancer hits approximately 2.8 million men in USA and the incidence rate raises with advanced years and in many countries [1]. Prostate cancer occurrence and mortality are up to 20-fold higher in industrialized countries respect to developing ones, thus for this purpose diet and lifestyle have been proposed as factors causing this discrepancy. Nutrition and exercise modify serum factors that slow down the growth and induce apoptosis in androgen-dependent PCa cells, while high levels of body mass index, blood pressure and several metabolic factors were correlated with high risk of prostate cancer death.

Many studies establish relevant ethnic diversity in death rates: Caribbean hold the highest rates in the world (26.3%), following Sub-Saharan Africans (10%) and, at last, the Asians show the lowest (2.5%). Perhaps genetic and environmental components contribute to generate this ethnic variety. Moreover, it was found that the risk of developing prostate cancer among American men, in particular African Americans, is highest than the other populations in the United States [2].

New therapies with established benefits on survival rate have been produced in last years [3–10], but nevertheless the gain in terms of survival is minimal. Prostate cancer is a very heterogeneous tumor which can show up indolent or very aggressive, usually with metastasis to other organs and bone [11–14], thus generating morbidity and mortality [15]. Prostate cancer can be clinically diagnosed as local or advanced and different treatments including surveillance, radiotherapy [16–18], radical prostatectomy and androgen-deprivation treatment are expected.

First line therapeutic options for local prostate cancer include different approaches: surgical prostatectomy, neoadjuvant therapy and radiation therapy. At present, the only advantageous treatment in PCa is surgical prostatectomy, but it involves adverse effects. Therefore, several studies recommended neoadjuvant therapy to improve survival rate. The mentioned therapy, known as androgen deprivation therapy (ADT), leads a reduction of androgens in prostate cancer cells of patients with advanced asymptomatic disease. It involves the use of drugs such as luteinising hormone-releasing hormone (LHRH) agonists, LHRH antagonists, antiandrogens, maximum androgen blockade and intermittent androgen deprivation. Radiation therapy is the main local disease treatment after surgical approach, in particular the hormonal therapy and radiotherapy combination improved survival rate [19]. On the contrary, treatment of choice of advanced prostate cancer is founded on chemotherapy: docetaxel in combination with prednisone, abiraterone acetate (AA) with prednisone, enzalutamide and cabazitaxel. Nevertheless, advanced prostate cancer remains an incurable disease and new alternative treatment are required.

The main screening exams to diagnose prostate cancer include digital rectal exam (DRE), serum level of prostate-specific antigen (PSA) and transrectal ultrasound guided biopsy. A suspected DRE alone reveals PCa, regardless of psa level, in about 18% of all patients; moreover, a suspected digital rectal exam has a positive predictive value of 5–30% in patients who have a PSA level up to 2 ng/ml. At present, measurement of PSA is the most usual marker able to correlate with prostate cancer risk, aggressiveness and outcome in order to detect prostate cancer [20]. Anyway, it has been found that several patients develop this type of cancer in spite of low PSA levels though a higher PSA level shows the presence of PCa [21–22]. Contrariwise, several factors as well as benign prostatic hyperplasia, gland inflammation and some drugs could arise from high PSA levels [23–26]. PSA screening is usually associated with over-diagnosis although it has decreased the death rate of PCa [27]. An important prognostic indicator of prostate cancer used to performe the histopathologic grading is Gleason score. Gleason-based grading allows to classify tumors according to their relative degree of differentiation by assigning a score from 1 (most differentiated) to 5 (least differentiated). Since it has been elucidated that tumors with similar histological patterns may provide different clinical outcomes, it became clear that Gleason score cannot exactly predict the aggressiveness of the disease although it is a powerful prognostic indicator [31–33]. The diagnosis of prostate cancer essentially results from cytologic or histopathologic confirmation, in particular in patients with PSA 4–10 ng/ml (“grey zone”). Hence, sometimes over-diagnosis and over-therapy of patients undergoing surgical or pharmacological therapy without a real necessity occur. For this reason and for the molecular heterogeneity of prostate cancer [34], it is necessary to identify alternative prostate cancer biomarkers, such as miRNA, to expedite and improve cancer diagnosis, prognosis and response to treatment, that will open the way to personalized therapeutic strategy. The purpose of this review is to analyze the improvement made to our knowledge of the function of miRNA in PCa tumourigenesis and for discovering which miRNAs may be considered helpful serum biomarkers.

### MiRNAs biogenesis and function

Small endogenous non-coding RNA (18–25 nt) are known as miRNAs. They can prevent protein expression through cleavage of specific target mRNAs or through inhibition of their translation [35]. Their biogenesis begins in the nucleus: a primary RNA (pri-miRNA), previously transcribed by RNA polymerase II (Pol II), is identified and processed by RNase III Drosha-DGCR8 complex to obtain a miRNA precursor (pre-miRNA), that is carried into the cytoplasm by Exportin-5 and Ran-GTP61. The RNase III Dicer, coupled to the transactivation-response RNA-binding protein (TRBP) enzyme, cleaves the pre-miRNA into a mature double-stranded miRNA of about 22 nucleotides. Afterwards, the functional strand of the mature miRNA is loaded with Argonaute (AGO) proteins into the RISC (RNA induced silencing complex): here miRNA drives RISC to bind the 3' UTR of a mRNA target and resulting in either mRNA cleavage, translational repression or deadenylation. On the contrary, the not functional strand will be degraded. In mammals there are four AGO (AGO1-4) proteins that induce mRNA degradation by interaction with deadenylation factors and translational machinery. Besides the aforementioned canonical mechanisms of biogenesis of miRNAs other “non canonical” mechanisms are emerging where some passages are bypassed. Mirtrons are one of the first noncanonical miRNAs that during their production do not require Drosha [36–38]. A recent study identifies another group called “5′ capped pre-miRNAs” that contains a 7-methylguanosine as a cap that includes miR-320 and miR-484. In addition there is also a Dicer-independent maturation of which is part miR-451 that is directly embedded in AGO2 and subsequently cut by exoribonuclease PARN into a mature form of miR-451 [39–43]. With respect to the phase of nuclear export, a current study revealed that miR-320 needs exportin 1 instead of exportin-5. MiRNAs can act by binding either the 3' UTR of the mRNA target through imperfect complementarity or via multiple sites by inhibition of the mRNA interaction with ribosomal complex and translational machinery. Furthermore, the imperfect complementarity with target ensures that miRNAs have multiple intracellular targets that lead to amplificate the biological effects. The identification of specific targets of miRNAs has been very difficult due to limited complementarity between miRNAs and mRNA transcripts. Functional characterization of miRNAs depends strongly on identification of their specific mRNA binding partners [44]. Several tools for target prediction have been realized to discover miRNA targets and most of them are conceived to concurrently decrease the false-positive incidence and raise the accuracy of their findings [45]. For example, TargetScan [46–49], one of the most used, allows to search miRNA families across several species by miRNA name, gene name or from more to less conserved. TargetScan is easy to use, but only considers stringent seeds, and ignores many potential targets. Therefore, computational prediction of miRNA target sites is an efficient tool to understand the molecular mechanisms of miRNA-mediated interactions and plays a key role in miRNA-based therapeutics in clinic. It is not unexpected that miRNAs have been shown to be involved in almost all key cellular processes, such as proliferation, differentiation, migration, apoptosis, stemness maintenance and metabolism, because each miRNA can modulate the expression of multiple mRNAs and, therefore, each of them may be targeted by several different miRNAs [50]. The deregulation of miRNAs expression in malignant diseases is due to the apoptotic evasion that results in tumorigenesis and drug-resistance, in particular during the tumor initiation and progression where act as oncogenes or as tumor suppressors and that are related to apoptosis. Therapies based on miRNA are used not only alone but in combination with anti-cancer therapies. This may reduce the toxicity and reduce the tumor drug resistance. For this reason, the studies are directed to new molecular therapies that block oncogenic miRNAs and stimulate the expression of those tumor suppressor [51–55]. In order to inhibit miRNA oncogenes, various strategies for miRNA-based therapeutics have been studied: a direct method involves oligonucleotides or virus-based constructs to inhibit the oncogenic miRNAs expression and to restore the expression loss of tumor suppressor miRNA, whereas an indirect method includes drugs that regulate miRNAs expression through their transcripts [56]. In the context of direct strategies, antisense oligonucleotides, miRNA sponges, miRNA-Masking Antisense oligonucleotides Technology (miRNA-mask) and small RNA inhibitors are used to block oncogenic miRNA. The action of the antisense oligonucleotides as competitive inhibitors is carried out through annealing to the target miRNA guide and inducing degradation or duplex formation. These oligonucleotides have been modified in their chemical structure to increase their stability, specificity and affinity binding. The most common changes include the insertion of 2′-O- methoxyethyl groups (2′-MOE), the 2′-O- methyl groups and locked nucleic acid (LNA) [57, 58]. Locked nucleic acid (LNA) is a modified RNA oligonucleotide, whose ribose moiety is structurally linked to an extra bridge connecting the 2′-O and the 4′-C atoms. This bridge “blocks” the ribose in the 3′-endo conformation, which is peculiar of the A-form duplexes. LNA oligonucleotides have a big ability to hybridize complementary single- or double-stranded DNA and complementary single-stranded RNA [59]. MiRNA sponges have been developed as new oligonucleotide constructs containing multiple complementary miRNA binding sites to the target miRNA. A repression of miRNA targets was observed when introduced into the cell and it seems to mimic an *in vitro* silencing of miRNAs. A bulge at the position normally cleaved by Argonaute 2 was introduced to increase the affinity of these decoy transcripts, thus allowing strong association of miRNA sponges with ribonucleoproteins of the corresponding miRNA [60]. A novel approach provides the use of the MiR-mask: 2′-O-methyl-modified antisense oligonucleotides with complementarity to the 3′ UTR target mRNA able to compete with endogenous miRNAs for own target. MiR-Mask protects the miRNA binding site in order to inhibit its target gene (mRNA) and, thereby, has the ability to repress oncogenic miRNA injurious functions at the target level [61]. MiRNA expression is regulated at transcriptional level by small molecule miRNA inhibitors. The chance to use synthetic miRNAs (called miRNA mimics) or viral constructs containing genes coding for miRNAs, such as the adeno associated viral vectors, is helpful to avoid the loss or decrease of a tumour suppressor miRNA [62–64]. Several investigations show that miRNAs could be considered excellent biomarkers for cancer diagnosis, prognosis and therapy. In cancer cells the over-expressed miRNAs that promote carcinogenesis by inhibition of tumor suppressor genes are considered oncogenic miRNAs (OncomiRs), while the down-expressed ones, that normally avoid cancer development by inhibition of proto-oncogenes expression, are tumor suppressor miRNAs [65].

Disease-specific miRNA expression profiles in different type of human cancers have been detected and identified by many reports [66–69]. The utility in diagnosis, staging, progression, prognosis and response to treatment makes miRNA expression profiling of human cancers still more important. However, the study of miRNAs is constantly changing because a better comprehension of the molecular mechanisms underlying their biogenesis and function will enable the development of new therapeutic miRNA-based techniques.

### MiRNAs as useful biomarkers in prostate cancer diagnosis and prognosis

MiRNAs represent an interesting target for biomarker discovery. Every miRNA can interact with various cellular pathways, and therefore alterations in the expression of few miRNAs may imply a bad regulation of several cellular processes conforming to the cancer complexity.

The issue of early cancer detection represents a promising field for microRNA-based diagnostics due to the currently lack of early detection methods and screening tests in many types of cancer.

In last years, it has been demonstrated through clinical and experimental discoveries that microRNAs have different expression in normal and cancer cells according to specific tissue expression signature [70]; microRNAs can also influence all hallmarks of cancer either by promoting or suppressing tumour development and progression [71]. Next to their intracellular functions, microRNAs have been detected as circulating molecules in many bio-organic fluids (urine, blood, saliva etc). Current investigations suggest that microRNAs are not only passively circulating molecules as byproducts, but also can act as mediators of intercellular communication via exosome-mediated transport in an ‘hormone-like’ way, thus mimicking the endocrine pathway [72]. Relying on these features, microRNAs could be considered excellent biomarkers. The big advantage they have is related to their high chemical stability in fresh or even formalin-fixed tissues and body fluids, so their potential as diagnostic markers increases in comparison with long noncoding RNAs or longer messenger RNAs and makes miRNA levels well suitable for testing in patient samples [73].

Several approaches to study miRNA profiling have been carried out [74]. It might be useful to identify a miRNAs panel testable in serum or tissue in order to discriminate patients with local prostate cancer that will eventually progress and will likely undergone to aggressive treatment from those ones with more advanced disease that have higher chances to benefit from special therapies such as novel agents acting on androgen pathway.

**Figure 1:**
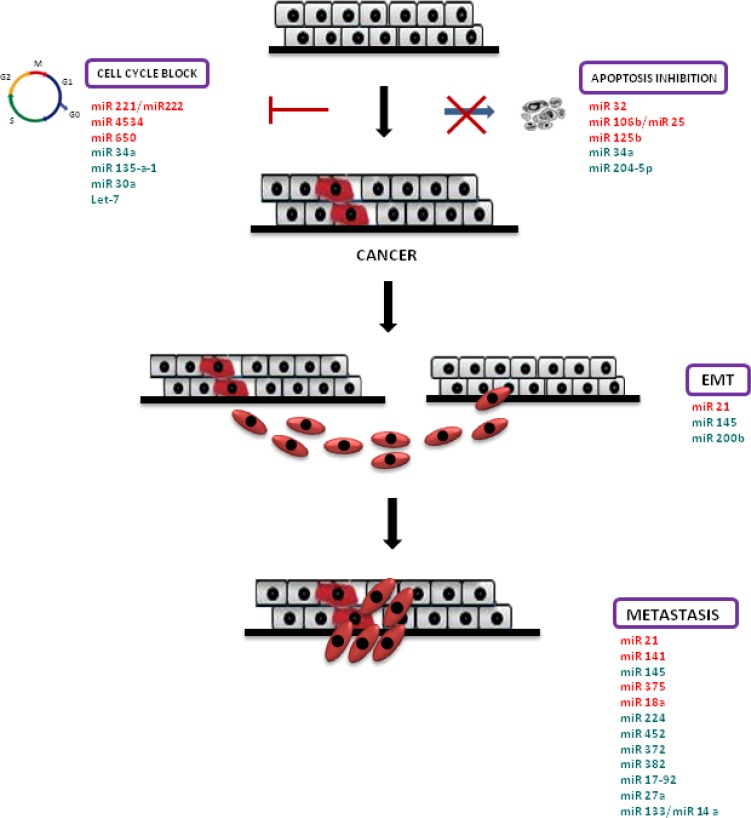
MiRNAs in PCa pathogenesis Schematic representation of prostate cancer pathogenesis and miRNAs involved in this process. Cell cycle block and apoptosis resistance lead to cancer; afterwards, prostate cancer cells are subjected to epithelial mesenchymal transition (EMT), which confers increased migratory capacity and invasiveness to generate metastases. Over-expressed miRNAs are highlighted in red, down-expressed miRNAs are highlighted in green.

MiRNA profiling on serum is really exciting because it can be carried out in a non-invasive way, and it allows to establish a diagnostic serum miRNA panel that would probably prevent prostatic biopsies in patients with high PSA levels, some of whom at last will not have PCa. To date, the attempt to define a clear miRNA profile in prostate cancer has proved inconclusive. Numerous conflicting outcomes regarding miRNAs expression in prostate cancer still raise. Several literature studies reported the opposite expression of some miRNA in this disease: some studies establish an overall down-expression of miRNAs in tumours, while other studies prove a general over-expression of miRs in cancer [75] (Figure 1).

### Oncogenic MiRNAs

One of the most common over-expressed oncomiR in cancer is miR-21. It controls the expression of many mRNA targets related to micro-vascular proliferation and tumor invasiveness. Its positive expression correlates with weak biochemical recurrence-free survival and has predictive value for biochemical recurrence risk in prostate cancer patients after radical prostatectomy [76]. Its expression is correlated with castration resistance and metastatic disease and enhances concurrently with clinical parameters (Gleason score, lymph node metastasis). Therefore, miR-21 is also helpful as biomarker to predict cancer progression [77]. Several literature studies showed that miR-221/miR-222 are increased in prostate cancer and that they could increase S-phase kinase associated protein 2 (Skp2), cyclin A and cyclin D1 via targeting p27Kip1 suppression, leading to cell cycle progression at G1- to -S phase, increased clonogenicity *in vitro* and enhanced tumorigenicity *in vivo* [78–80]. To date, the miR-141 function in PCa is not clear, but it has been revealed that it is over-expressed after castration, thus it's likely its involvement in androgen mechanism, both in androgen-dependent and in metastatic castration-resistant prostate cancer. For this reason, the role of miR-141 in cancer progression seems interesting [81]. However, miRNAs expression in body fluids, in particular in serum, allows to obtain many interesting results concerning their role. In 2008, it was demonstrated for the first time by Mitchell et al. that cancer associated miRNAs can be easily detected and measured in biological fluids and that miR-141 levels were higher in serum of PCa patients [82].

In this context, miR-141 could be considered an important tool for prostate cancer diagnosis and prognosis. In fact, it has been elucidated that discrimination among low, intermediate and high risk patients with PCa can be carried out by miRNAs [77]. According to a good study concerning circulating miRNAs, it has been established that a decreased expression in non metastatic patients was also showed by miR-375 and therefore it appeared really important for early diagnosis [83, 84]. miR-18a is strongly over-expressed in prostate cancer tissues, thus acting as oncogenic miRNA. Its expression is really increased in peripheral blood of patients with prostate cancer compared with benign prostatic hyperplasia (BPH) patients and healthy individuals, as showed by Al-Kafaji G et al.

In addition, they have found that an higher miR-18a expression level correlates with PCa progression. These studies indicate that peripheral blood oncogenic miR-18a can function as a new potential non-invasive biomarker for PCa and can also make easy the distinction between PCa and BPH [85]. Another oncogenic miRNA overexpressed in PCa and positively correlated with poor overall and PSA recurrence free survival is miR-4534. It is hypermethylated in normal cells and tissues compared to those of PCa and exerts its oncogenic effects partly by arresting the tumor suppressor PTEN gene [86]. Its overexpression induces pro-cancerous characteristics in non-cancer cell line whereas its knockdown impairs cell proliferation, migration/invasion and induces apoptosis and then G0/G1 cell cycle block in PCa [87]. High level of miR-650 was discovered in PCa samples. Oncogenic activity of miR-650 in PCa is explicated through suppression of cellular stress response 1 (CSR1) expression. It was showed that miR-650 inhibition reversed the suppressed expression of CSR1, repressed colony formation and blocked cell cycle entry to the S phase in DU145 and PC3 cells [88].

MiR-32 is highly expressed in castration resistant prostate cancer (CRPC) samples compared to benign prostatic hyperplasia samples. A study demonstrated that miR-32 exerts oncogenic characteristics by targeting on both B-cell translocation gene 2 (BTG-2) and phosphoinositide-3-kinase interacting protein 1 (PIK3IP1), which controls the inhibition of PI3K, a well-known regulator of cell proliferation, migration and survival [89]. MiR-106b/miR-25 cluster is an oncomir cluster, identified by computational approach, that is found associated with prostate cancer progression by targeting CASP7 (caspase 7, apoptosis related cysteine peptidase) mRNA, which is down-expressed both in primary prostate cancer and metastatic lesions [90]. MiR-125b is an androgen receptor-induced miRNA. Its induction in prostate cancer cell line LNCaP inhibits apoptosis and enhances cell proliferation. It was demonstrated that miR-125b activates the p53 network by interrupting Mdm2 degradation via targeting p14^ARF^, which mediates the Mdm2 sequestration [91]. A summary of several oncogenic miRNAs found in prostate cancer is included in Table 1.

**Table 1:** Some of oncogenic MiRNAs involved in prostate cancer

Oncogenic miRNAs	Function	Reference
**miR-21**	promotes tumor invasiveness and induces castration-resistance phenotype.	[76–77]
**miR-221/miR-222**	enhance cell proliferation, invasion, cell survival, increase clonogenicity and enhance tumorigenicity *in vivo*.	[78–80]
**miR-141**	is important in androgen-dependent and in metastatic castration-resistant.	[81–82]
**miR-375**	is important for an early diagnosis.	[83–84]
**miR-18a**	promotes cancer progression.	[85]
**miR-4534**	induces pro-cancerous characteristics in non-cancer cell line.	[87]
**miR-650**	suppresses the cellular stress response 1 (CSR1) expression.	[88]
**miR-32**	inhibits apoptosis and enhances proliferation.	[89]
**miR-106/miR-25**	facilitate tumor progression.	[90]
**miR-125b**	enhances cell proliferation and inhibits apoptosis.	[91]

### Tumour suppressor MiRNAs

The involvement of miR-34 family as tumour suppressor in cancer has been reported by several investigations and many strategies to replace miR-34 have been developed in order to treat cancer [92, 93]. MiR-34a may exert its tumor suppressor role via targeting several signaling molecules involved in various stages of PCa progression. DNA damage and oncogenic stress strongly induced miR-34 expression in a p53-dependent manner. The distinctive features of p53 activity are promoted by miR-34 activation [94–95]: induction of cell cycle block and apoptosis through down-modulation of different proteins (CDK4, CDK6, cyclin D1, cyclin E2, E2F3 and BCL2) [96–98].

MiR-145 is a tumor suppressor and its expression is controlled by p53 pathway [99]. MiR-145 has been found down-expressed in breast cancer and colorectal cancer [100, 101]. Avgeris et al. found that higher risk for biochemical recurrence and significantly decreased disease-free survival were caused by miR-145 down-expression and that its decrease in prostate cancer was related to higher Gleason scores, advanced clinical stage, greater tumor diameter and higher PSA levels during follow-up [102]. Recently, it was discovered that miR-224 leads to migration and invasion of prostate cancer cells by targeting a miR-224 target gene, called apelin, whose inhibited expression allows the miRNA to inhibit cell migration and invasion. Moreover, miR-224 down-expression correlates significantly with advanced clinical stage and metastasis [103]. Kristensen et al. elucidated that proliferation, migration and invasion in prostate cancer cell lines PC3 and DU145 were inhibited by miR-224 and miR-452 through blocking cell cycle, cellular adhesion and motility [104]. Although the miR-200 family can be regulated at post-transcriptional levels, Zheng Zhang et al. found that ERG directly over-expresses the tumour suppressive miR-200b subfamily in human prostate tumours. In particular ERG can directly bind to miR-200b/a/429 promoter to facilitate its transcription in prostate cancer cells [105]. Tumour suppressor miR-382 represents an important new mechanism for understanding prostate cancer. It suppresses PCa cell proliferation, migration, invasion and metastasis through inhibiting COUP-TFII, including Snail and matrix metalloproteinase 2 (MMP2); in contrast, miR-382 suppression exhibited an opposite effect [106]. miR-372 has the ability to inhibit proliferation activity, migration, and invasion of DU145 cell line. One confirmed target of miR-372 is p65, whose knocked down expression induced a decrease in proliferation activity, migration and invasion. In both these processes CDK8, MMP-9, and prostate-specific antigen were involved [107]. miR-17-92a cluster miRNAs arises from a polycistronic transcription unit C13orf25 that generates six mature miRNAs: miR-17, miR-18a, miR-19a, miR-19b, miR-20a and miR-92a. These cluster miRNAs have a therapeutic benefit for both androgen-independent and -dependent PCa cells. Their expression are reduced in cancerous prostate tissues and also in aggressive prostate cancer cells. Restoration of expression of all members of miR-17-92a cluster showed depressed expression of cell cycle regulatory proteins cyclin D1 and SSH1 and inhibited EMT by reducing cell migration and mesenchymal markers expression and by increasing surface localization and expression of the E-Cadherin. Moreover, this cluster improves sensitivity of LNCaP androgen dependent prostate cancer cells to anti-androgen drug Casodex, AKT inhibitor MK-2206 2HCl and docetaxel [108]. miR-27a functions as tumour suppressor in PCa cell by repression of MAP2K4 which works as an oncogene in PCa cell lines [109]. Xu B et al. found that hsa-miR-135a-1 exerts its action as a potential tumour suppressor in metastatic PCa by targeting EGFR [110]. Human tumour suppressor miRNA-204-5p facilitates apoptosis by BCL2 in PCa cells. In miR-204-5p-transfected cells BCL2 mRNA and protein expression decreased and consequently cytochrome C delivery from mitochondria and caspase 3 and caspase 3/7 activation were induced [111]. Zhang et al. suggest that miR-30a may function as a novel tumour suppressor in castration-resistant prostate cancer (CRPC) tissues. Its anti-oncogenic activity may occur by the reduced expression of a distinct cell cycle protein, cyclin E2 (CCNE2) [112]. Let-7 gene encodes a highly conserved miRNA family, which are significantly down-expressed in localized prostate cancer. Let-7 has been shown to target oncogenes implicated in cell-cycle progression, cell proliferation, migration, differentiation and epithelial-to-mesenchymal transition (EMT) progression. Let-7 miRNA family exerts tumour suppressor characteristics via targeting multiple oncogenes including RAS, HMGA2, Ezh2, Lin28 and c-Myc [113]. Thus, loss of let-7 miRNAs correlates with prostate cancer progression [114]. It has been discovered that miR-133 and miR-146a suppress prostate cancer tumor progression via targeting EGFR, a tumour promoter for this disease. Thus, loss of miR-133 and miR-146a may be attributed to enhancement of EGFR signaling, leading to aggressive prostate cancer progression [115]. A summary of some tumor suppressor miRNAs in prostate cancer is included in Table 2.

**Table 2:** Some of suppressive miRNAs involved in prostate cancer

Tumour suppressor miRNAs	Function	Reference
**miR-34a**	induces cell-cycle arrest, cell senescence and apoptosis and inhibits cell proliferation and cell invasion.	[96–98]
**miR-145**	inhibits invasion, migration and arrests cell cycle.	[99–102]
**miR-224**	inhibits invasion and migration of PCa cells.	[103]
**miR-452**	regulates cell cycle, cellular adhesion and motility.	[104]
**miR-200b**	inhibits PCa cell growth and invasion.	[105]
**miR-382**	inhibits PCa cell proliferation, migration, invasion and metastasis.	[106]
**miR-372**	inhibits proliferation, migration and invasion of DU145 cells.	[107]
**miR-17-92a**	decreases cell cycle regulatory proteins and the expression of mesenchymal markers.	[108]
**miR-27a**	suppresses MAP2K4 in PCa cell.	[109]
**has-miR-135-a-1**	inhibits cell growth, cell cycle progression, migration, invasion, and xenograft tumor formation.	[110]
**miR-204-5p**	promotes apoptosis by targeting BCL2 in PCa cell.	[111]
**miR-30a**	reduces expression of cell cycle protein, cyclin E2.	[112]
**let-7 miRNAs**	regulate cell cycle, cell migration, cell proliferation and epithelial-to-mesenchymal transition progression.	[113–114]
**miR-133/miR-146a**	suppress tumor progression via targeting EGFR.	[115]

## CONCLUSIONS

Prostate cancer is the secondary highest cause of malignancy mortality in male. It is estimated, in 2016, about two hundred thousand men will be diagnosed with PCa, and about twenty seven thousand will die of PCa. In this context it becomes extremely important to improve early diagnosis and treatment to reduce cancer-related mortality. In PCa there is a deregulation of several miRNAs that may act as oncogenes or tumour suppressors. The miRNA profiling studies demonstrate that mRNAs may act independently or in partnership with other transcription factors to regulate gene transcription, which ultimately leads to perturbed cellular processes in PCa. The knowledge of miRNAs biology, the study of their functions and molecular mechanisms in recent years are growing more and more. They are strongly involved in cancer development, in particular several miRNAs are implicated in cell transformation for the development of cancer cells, metastasis and treatment resistance. Many studies established that specific miRNAs are over-expressed or down-expressed concurrently in different types of human cancer and often related to specific cytogenetic abnormalities.

The most important strategies for miRNA-based therapeutics consist of a direct and an indirect method. Direct strategies include oligonucleotides or virus-based constructs in order to block the oncogenic miRNAs expression, while the indirect ones involve drugs to regulate miRNAs expression. Understanding the specific roles of some miRNAs and their involvement in prostate cancer development and progression could lead to improve the course of this cancer and could open new therapeutic options in prostate cancer treatment. Many discoveries have demonstrated that miRNAs are helpful tools to make diagnosis and prognosis of prostate cancer and can predict the clinical outcome of patients. To date, miRNAs can be evaluated using cheap technologies in serum of patients. Therefore, the development of easy kits to detect circulating miRNAs by nanotechnologic approach is necessary to provide the diffusion of these biomarkers in the national health system and could represent an important step forward in the field of molecular therapies [116].
